# El'gygytgyn impact crater, Chukotka, Arctic Russia: Impact cratering aspects of the 2009 ICDP drilling project

**DOI:** 10.1111/maps.12146

**Published:** 2013-07-02

**Authors:** Christian Koeberl, Lidia Pittarello, Wolf Uwe Reimold, Ulli Raschke, Julie Brigham-Grette, Martin Melles, Pavel Minyuk, John Spray

**Affiliations:** 1 Department of Lithospheric Research, University of ViennaAlthanstrasse 14, A-1090, Vienna, Austria; 2 Natural History MuseumBurgring 7, A-1010, Vienna, Austria; 3 Museum für NaturkundeInvalidenstrasse 43, 10115, Berlin, Germany; 4 Humboldt-Universität zu BerlinUnter den Linden 6, 10099, Berlin, Germany; 5 Department of Geosciences, University of MassachusettsAmherst, Massachusetts, 01003, USA; 6 Institute of Geology and Mineralogy, University of CologneZuelpicher Strasse 49a, D-50674, Cologne, Germany; 7 North-East Interdisciplinary Scientific Research Institute, Far East Branch – Russian Academy of Sciences16 Portovaya St., 685000, Magadan, Russia

## Abstract

The El'gygytgyn impact structure in Chukutka, Arctic Russia, is the only impact crater currently known on Earth that was formed in mostly acid volcanic rocks (mainly of rhyolitic, with some andesitic and dacitic, compositions). In addition, because of its depth, it has provided an excellent sediment trap that records paleoclimatic information for the 3.6 Myr since its formation. For these two main reasons, because of the importance for impact and paleoclimate research, El'gygytgyn was the subject of an International Continental Scientific Drilling Program (ICDP) drilling project in 2009. During this project, which, due to its logistical and financial challenges, took almost a decade to come to fruition, a total of 642.3 m of drill core was recovered at two sites, from four holes. The obtained material included sedimentary and impactite rocks. In terms of impactites, which were recovered from 316.08 to 517.30 m depth below lake bottom (mblb), three main parts of that core segment were identified: from 316 to 390 mblb polymict lithic impact breccia, mostly suevite, with volcanic and impact melt clasts that locally contain shocked minerals, in a fine-grained clastic matrix; from 385 to 423 mblb, a brecciated sequence of volcanic rocks including both felsic and mafic (basalt) members; and from 423 to 517 mblb, a greenish rhyodacitic ignimbrite (mostly monomict breccia). The uppermost impactite (316–328 mblb) contains lacustrine sediment mixed with impact-affected components. Over the whole length of the impactite core, the abundance of shock features decreases rapidly from the top to the bottom of the studied core section. The distinction between original volcanic melt fragments and those that formed later as the result of the impact event posed major problems in the study of these rocks. The sequence that contains fairly unambiguous evidence of impact melt (which is not very abundant anyway, usually less than a few volume%) is only about 75 m thick. The reason for this rather thin fallback impactite sequence may be the location of the drill core on an elevated part of the central uplift. A general lack of large coherent melt bodies is evident, similar to that found at the similarly sized Bosumtwi impact crater in Ghana that, however, was formed in a target composed of a thin layer of sediment above crystalline rocks.

## Introduction

**Fig. 1:**
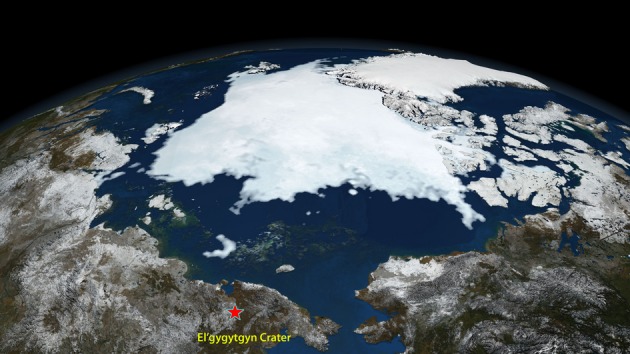
Extent of the Arctic sea ice in the summer of 2008 (NASA Goddard Space Flight Center image). The location of the El'gygytgyn structure in the northeastern corner of Siberia, at the Chukotka Peninsula, is also shown. The crater is at a crucial place with respect to the Arctic ice cover, and the study of the lake sediments, which provide valuable information on the development of the climate in the area during the past approximately 3.5 Myr, was a major driving force of the drilling project.

The El'gygytgyn impact structure is located in the far northeastern part of Russia (centered at 67°30′ N and 172°05′ E), on the Chukotka peninsula (Fig. 1). El'gygytgyn consists of a circular depression with a rim diameter of about 18 km that is filled by a lake with a diameter of 12 km that is off-center with regard to the crater. The structure was discovered and described as a gigantic volcanic crater in 1933 (Obruchev 1957). The first suggestion that this structure might be of impact origin was made by Nekrasov and Raudonis (1963); these authors searched unsuccessfully for coesite in thin sections of volcanic rocks from the crater rim and, consequently, concluded that the “El'gygytgyn basin” had a tectonic and volcanic origin. Without any further evidence, this structure appeared in a list of probable terrestrial impact craters by Zotkin and Tsvetkov (1970). From a study of satellite imagery of the structure, Dietz and McHone (1976) suggested that El'gygytgyn might be the largest Quaternary impact crater preserved on Earth. Shortly afterward, Dietz (1977) suggested that El'gygytgyn might be the source crater of the Australasian tektites.

Gurov and co-authors visited the El'gygytgyn structure in 1977 and confirmed its impact origin after finding shock metamorphosed rocks and impact melt rock (Gurov et al. 1978; Gurov and Gurova 1979; Gurov et al. 1979). Investigations of the El'gygytgyn crater by these researchers continued into the 1980s and 1990s (Gurov and Gurova 1991). Further work was performed by Feldman et al. (1981), who gave a short description of the geology of the crater and its target. Gurov and colleagues studied the main types of impact melt rocks and highly shocked volcanic rocks. A preliminary geophysical investigation of the crater was carried out by Dabizha and Feldman (1982). The geological structure of the crater rim was described by Gurov and Gurova (1983) and Gurov and Yamnichenko (1995); see also Gurov et al. (2007). Although the impact origin of the El'gygytgyn structure had been recognized and confirmed more than 20 years ago, an endogenic origin for this structure was once again proposed later by Belyi (1982, 1998). Nevertheless, the matter is firmly settled due to the unambiguous evidence for an impact origin in the form of shock metamorphic effects in the crater rocks.

First age determinations for the El'gygytgyn impact crater were obtained by fission track (4.52 ± 0.11 Ma; Storzer and Wagner 1979) and K-Ar dating (3.50 ± 0.50 Ma; Gurov et al. 1979). These data quickly invalidated the suggestion of Dietz (1977) of El'gygytgyn as the source of the Australasian tektites (of 0.8 Ma age). More detailed fission track analyses resulted in an age for the crater of 3.45 ± 0.15 Ma (Komarov et al. 1983). Subsequently, Layer (2000) performed ^40^Ar-^39^Ar age dating of impact glasses and found an age of 3.58 ± 0.04 Ma for the impact event, in good agreement with some of the earlier results.

Here, we discuss the impact cratering-related aspects of a recent international and multidisciplinary scientific drilling project at El'gygytgyn that led to the recovery of a drill core through the lake sediments, impact breccia, and uplifted and brecciated bedrock near the crater center.

## Geological setting of the El'gygytgyn impact structure

Among the slightly more than 180 currently confirmed impact structures on Earth, there are just a few (Lonar, Logancha, Vista Alegre, Vargeão, and Cerro do Jarau) that formed within basaltic volcanic rock. However, a major aspect of the importance of El'gygytgyn is that it represents the only currently known impact structure formed in siliceous volcanic rocks, including tuffs. Thus, the impact melt rocks and target rocks provide an excellent opportunity to study shock metamorphism of silicic volcanic rocks. The shock-induced changes observed in porphyritic volcanic rocks from El'gygytgyn can be applied to a general classification of shock metamorphism of siliceous volcanic rocks.

At 18 km diameter, El'gygytgyn is a medium-sized impact structure. Even though the rim is partly eroded, especially in the southeastern part, the rim height is generally about 180 m above the lake level and 140 m above the surrounding area. An outer ring feature, on average 14 m high, occurs at about 1.75 crater radii from the center of the structure. A similar outer ring structure was noted at the Bosumtwi impact structure (e.g., Koeberl and Reimold 2005 and references therein), but the nature and origin of such features have yet to be explained. The El'gygytgyn crater is surrounded by a complex system of radial and concentric faults. The density of the faults decreases from the bottom of the rim to the rim crest and outside the crater to a distance of about 2.7 crater radii (Gurov et al. 2007).

**Fig. 2:**
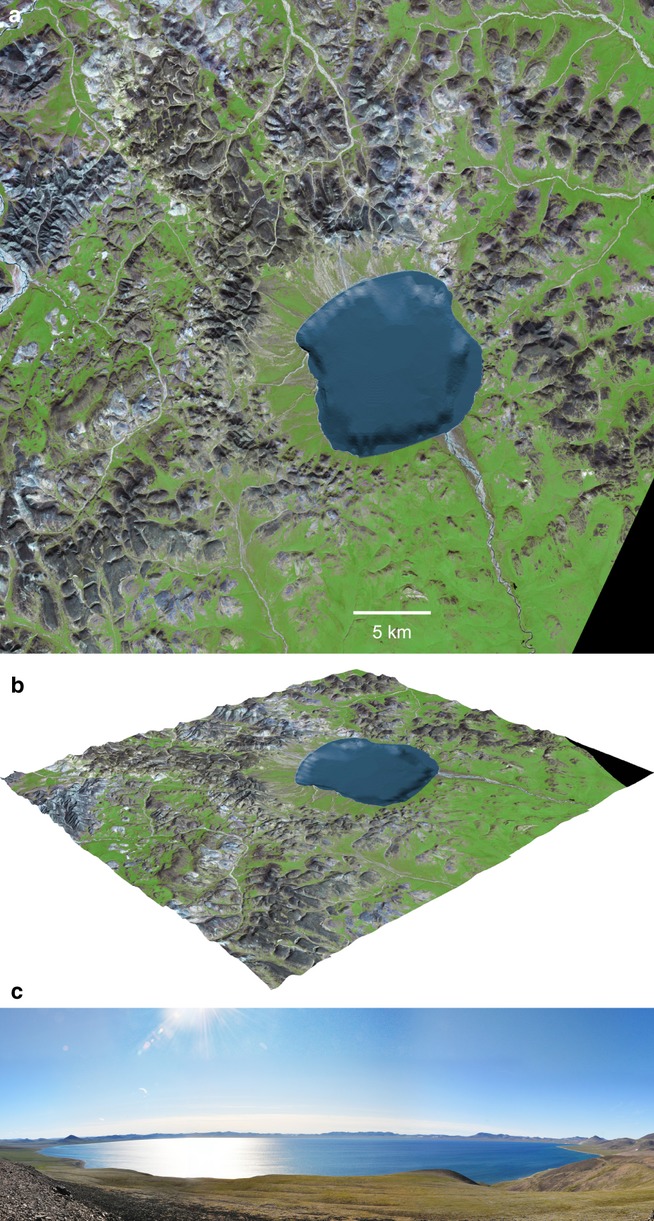
a) Satellite image of the El'gygytgyn impact crater, Arctic Russia (NASA Aster image). The image shows the 12 km-diameter Lake El'gygytgyn, which is asymmetrically located with the 18 km-diameter impact crater. b) Perspective view of satellite image with digital elevation model (DEM); projection by M. Schiegl (Austrian Geological Survey), and DEM of Lake El'gygytgyn from digital elevation model data by M. Nolan (University of Alaska at Fairbanks) at: http://www.uaf.edu/water/faculty/nolan/lakee/data.htm (accessed 2009). c) Panoramic image of El'gygytgyn crater and lake; view from the northeast to the southeast (U. Raschke, July 2011).

The crater and its lake are shown in Figs. 2a and 2b. The lake that fills part of the crater interior has a maximum depth of about 170 m and is surrounded by a number of lacustrine terraces (cf. Gurov et al. 2007). Only minor remnants are preserved of the highest terraces that are about 80 and 60 m above the present-day lake level. The widest terraces are 40 m above the current lake level and surround the lake on the west and northwest sides; the most modern terrace is 1–3 m above the current lake level, indicating severe changes in the water level with time. Even though many small creeks discharge into the lake, the only outlet is the Enmivaam River, which cuts the crater rim in the southeast.

A central peak is not exposed on the recent surface of the crater floor, nor is it evident in bathymetric data of the lake bottom. However, from gravity measurements, Dabizha and Feldman (1982) suggested the presence of an approximately 2 km wide central peak underneath postimpact sediments, and centered relative to the crater outline. Nolan et al. (2003) suggested that the central uplift is centered within the outline of the lake, which, however, would offset the central uplift relative to the crater center. Seismic investigations during the preparation of the drilling project revealed the presence of a buried central uplift, not unlike the situation at the Bosumtwi impact structure in Ghana, with a diameter of approximately 2 km, and which is centered with respect to the crater rim rather than the lake outline (Gebhardt et al. 2006). According to these seismic measurements, the thickness of the sedimentary fill near the crater center (above and near the central uplift) is about 360–420 m. The sediments are underlain by units with distinctly higher seismic velocities that were interpreted as allochthonous breccia, 100–400 m thick (Gebhardt et al. 2006; Niessen et al. 2007).

In terms of regional geology, the crater is excavated in the outer zone of the Late Cretaceous Okhotsk-Chukotka Volcanic Belt (OCVB), mainly involving the so-called Pykarvaam Series (88.5 ± 1.7 Ma; Stone et al. 2009). Laser ^40^Ar/^39^Ar dating of the unshocked volcanic rocks in the crater yielded an age-range from 89.3 to 83.2 Ma (Layer 2000). The volcanic sequence includes lava, tuffs, and ignimbrites of rhyolitic to dacitic composition, which belong to the younger Voronín and Koekvun FM. Rarely, andesites and andesitic tuffs occur. The whole sequence is, in general, gently dipping at 6° to 10° to the east-southeast (Gurov and Koeberl 2004). Detailed field observations by Gurov and co-workers (Gurov and Koeberl 2004) in the 1990s allowed establishing a rough pre-impact stratigraphy. From the top to the bottom, it consists of approximately 250 m of rhyolitic ignimbrites, approximately 200 m of rhyolitic tuffs and lavas, approximately 70 m of andesitic tuffs and lavas, and approximately 100 m of rhyolitic to dacitic ash and welded tuffs. This sequence dominates in the southern, western, and northern part of the crater, whilst the southeastern and eastern parts of the crater mainly consist of dacitic and andesitic lavas. A basalt plateau, approximately 110 m in thickness, overlies the rhyolites and ignimbrites in the northeastern part of the crater rim (Gurov and Koeberl 2004). These basalts possibly belong to the Koekvun volcanic suite, which is located above the Pykarvaam series in the volcanic sequence (83.1 ± 0.4 Ma; Stone et al. 2009).

**Fig. 3:**
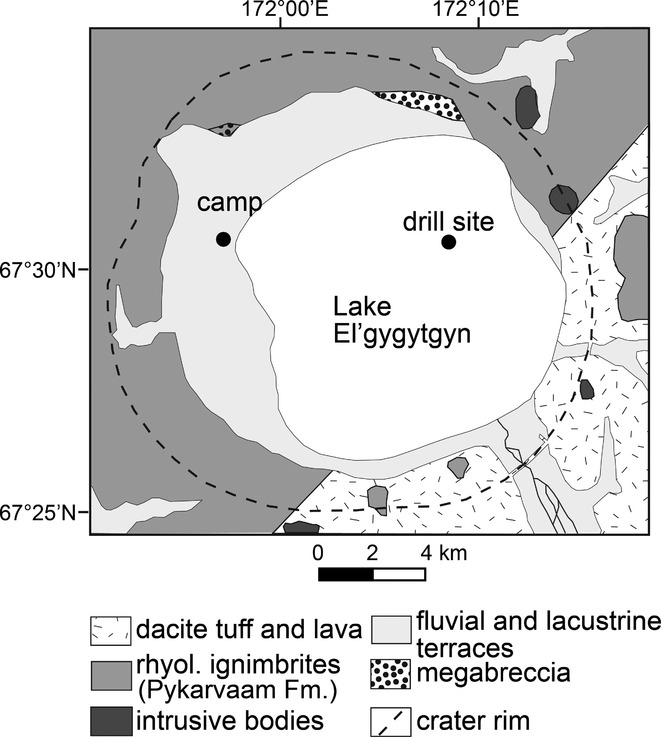
Simplified geological map of the El'gygytgyn area (modified after Gurov and Koeberl 2004; Gurov et al. 2005; and Stone et al. 2009). The figure also shows the location of the drill rig and the camp site for the ICDP project, and the two drilling locations (black dots).

The general geology at El'gygytgyn is summarized in Fig. 3. The most widespread lithology represents pyroclastic deposits of rhyolitic-dacitic composition (approximately 89% by volume). Occurrences of basaltic rock are limited to isolated patches. In terms of mineralogy, the general composition of the target is dominated by quartz clasts and grains, K-feldspar (Or_60–80_), plagioclase (An_20–30_), biotite, and rarely amphibole, embedded in a fine-grained clastic matrix with glass, quartz, and feldspar fragments. The fabric of the matrix ranges from glassy to fine-grained granular, occasionally with spherulites (Gurov et al. 2005). The less abundant andesites and andesite tuffs occur only locally and contain fragments and clasts of andesine (An_45_ to An_40_), clinopyroxene, and amphibole (Gurov and Koeberl 2004).

On the surface, impact melt rocks occur at El'gygytgyn mainly in the form of redeposited material on the lacustrine terraces. No actual outcrops of impact breccias have been found so far. The most probable origin of these rocks is from the ejecta blanket and fallback material that is now only present as eroded remnants and material that slumped off the rim. The impact melt rocks include aerodynamically shaped glass bombs and shock metamorphosed breccias. The glass bombs are generally fresh and do not display significant postimpact hydrothermal alteration or alteration due to weathering (Gurov and Koeberl 2004; Gurov et al. 2005).

## Rationale for the Drilling Project

Drilling allows obtaining information on the subsurface structure of impact craters, provides ground-truth for geophysical studies, and delivers samples of rock types not exposed at the surface. For more than a decade, the International Continental Scientific Drilling Program (ICDP) has supported projects to study impact craters (Koeberl and Milkereit 2007). The first ICDP study of an impact structure was at the subsurface Chicxulub impact crater, Mexico, from late 2001, which reached a depth of 1511 m and intersected 100 m of impact melt breccia and suevite. Between June and October 2004, the 10.5 km Bosumtwi crater, Ghana, was drilled with ICDP support. It is a well preserved complex impact structure with a pronounced rim and is almost completely filled by the 8 km diameter Lake Bosumtwi. This is a closed-basin lake that has wide paleoclimatic significance and allowed researchers to accumulate a detailed paleo-environmental record. In terms of impact studies, Bosumtwi is one of the best preserved young complex craters known, and is the source crater of the Ivory Coast tektites. The drilling outcomes also allowed correlating all the geophysical studies, and provide material for geochemical and petrographic correlation studies between basement rocks and crater fill in comparison with tektites and ejected material. Sixteen different cores were drilled at six locations within the lake, to a maximum depth of 540 m. Borehole logging as well as vertical seismic profiling (to obtain 3-D images of the crater subsurface) were performed in the two deep boreholes. About 2.2 km of core material was obtained. This includes approximately 1.8 km of lake sediments and 0.4 km of impactites and fractured crater basement (in the deep crater moat, and on the central uplift). For details of the Bosumtwi drilling project, see Koeberl et al. (2007a). Chesapeake Bay, a much larger impact structure than Bosumtwi or El'gygytgyn, was drilled to a depth of almost 2 km in 2005–6; results of this drilling project are reported by, e.g., Gohn et al. (2008, 2009).

The El'gygytgyn impact crater is a unique study target for an ICDP project for two main reasons: (1) predrilling site surveys indicated that a full-length sediment core would yield a complete record of climate evolution for the past 3.6 Myr in an area of the high Arctic for which few paleoclimate data exist, and (2) it is the only known impact crater on Earth that has formed in acidic volcanic rocks, allowing the study of shock metamorphic effects in such target rocks and the geochemistry and petrology of “volcanic” impactites, and potential analog studies for other planets. These aspects clearly mark El'gygytgyn as a world-class research site. As at Bosumtwi, the deep basin that formed as a result of the impact event is an ideal location for the accumulation of lake sediments that carry paleoclimate information.

Its sedimentological aspect makes Lake El'gygytgyn unique in the terrestrial Arctic, especially because geomorphological evidence from the catchment has suggested that the crater was never completely glaciated throughout the Late Cenozoic. Two sediment cores retrieved from the deepest part of the lake in 1998 and 2003 revealed lacustrine basal ages of approximately 250 and 340 ka, respectively, and thus, represent the longest continuous climate records available at that time from the Arctic region. The continuous sedimentation confirmed the lack of glacial erosion, and the sediment composition underlined the sensitivity of this lacustrine environment to reflect high-resolution climatic change on Milankovitch and sub-Milankovitch time scales (cf. Brigham-Grette et al. 2007).

Seismic investigation carried out during expeditions in 2000 and 2003 led to a depth-velocity model of brecciated bedrock overlain by a different breccia layer, in turn overlain by two lacustrine sedimentary units of up to 350 m thickness (e.g., Niessen et al. 2007). The upper well-stratified sediment unit appears undisturbed apart from intercalation with debris flows near the crater wall. Extrapolation of sedimentation rates obtained from earlier shallow cores indicated that the entire Quaternary and possibly beyond was expected to be represented in the 170 m thick upper unit; the lower unit, which was probably characterized by a higher sedimentation rate, covered the earlier postimpact history of the lake.

In terms of impact research, El'gygytgyn gains its importance by being the only currently known impact structure formed in siliceous volcanic rocks, as mentioned above. The shock-induced changes observed in porphyritic volcanic rocks from El'gygytgyn can be applied to a general classification of shock metamorphism of siliceous volcanic rocks (cf. Gurov et al. 2005). However, impactites exposed on the surface have been almost totally removed by erosion, and thus the deep drilling project provides a unique opportunity to study the crater-fill impactites in situ and determine their relations and succession. The goals of the project included, inter alia, obtaining information on the shock behavior of the volcanic target rocks, the nature and composition of the asteroid that formed the crater, and the abundance of impact melt rocks.

Main coring objectives included to obtain replicate cores of 630 m length to retrieve a continuous paleoclimate record from the deepest part of the lake and information about the underlying impact breccias and bedrock. Studies of the impact rocks offer the planetary community the opportunity to study a well-preserved crater uniquely situated in igneous volcanic rocks. An additional shorter core was to be drilled into permafrost from the adjacent catchment to test ideas about Arctic permafrost history and sediment supply to the lake since the time of impact.

## Drilling Project and Operations

The El'gygytgyn drilling project took almost a decade from the first planning steps to execution. ICDP funded a workshop in Amherst MA, USA, in November of 2001 to stimulate scientific interests in deep drilling at Lake El'gygytgyn. A second workshop was held in March 2004 in Leipzig, Germany, to synthesize results from a 2003 expedition and discuss the possibilities for interdisciplinary research goals for drilling. After completion of presite surveys (cf. Melles et al. 2011), a pre-proposal was submitted to ICDP in January 2004, outlining the status of our science and planning efforts. A review of that pre-proposal by the ICDP Science Advisory Group (SAG) was very encouraging, and thus a full proposal was submitted in January 2005, which was well received and was accepted for funding (partial funding covering some of the drilling operations only) in the summer of 2005. The following years were occupied by intense fundraising efforts, which were necessary due to the final cost of about US$10 million for the entire drilling operations, and by putting the required complex technical and logistical requirements (including permitting issues) of the project in place. Finally, movement of equipment began in 2008, permafrost drilling was performed at the end of 2008, and sediment and impactite core drilling at the center of the frozen lake commenced in February of 2009 and was completed in May 2009.

**Fig. 4:**
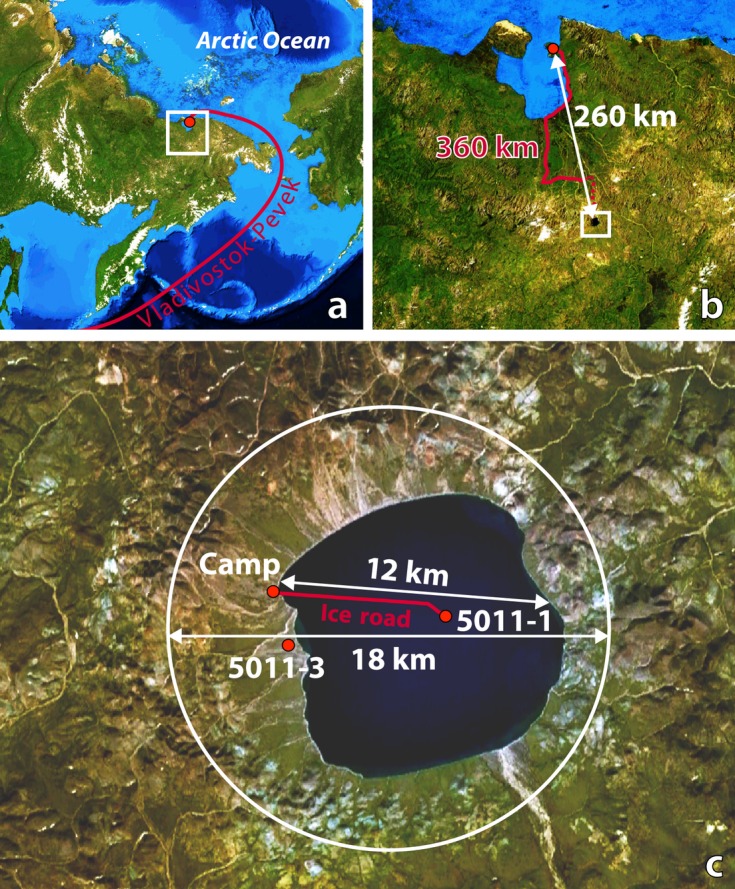
Location and setting of the El'gygytgyn impact structure with respect to the logistics of the drilling project (modified from Melles et al. 2011). a) Location of the crater in central Chukotka, NE Russia, about 850 km west of the Bering Strait. The drill rig and all equipment arrived at the lake first by barge from Vladivostok along the indicated route. b) All equipment was transported to the site from the town of Pevek, a gold mining center located on the coast of the East Siberian Sea. Helicopters were used to transport scientists, food, and delicate equipment out to the drill site, whereas the 17 shipping containers with the drilling system were transported by truck. c) Satellite image with lake and crater diameter, the locations of ICDP Sites 5011-1 and 5011-3, and the outline of crater rim (white circle).

**Fig. 5:**
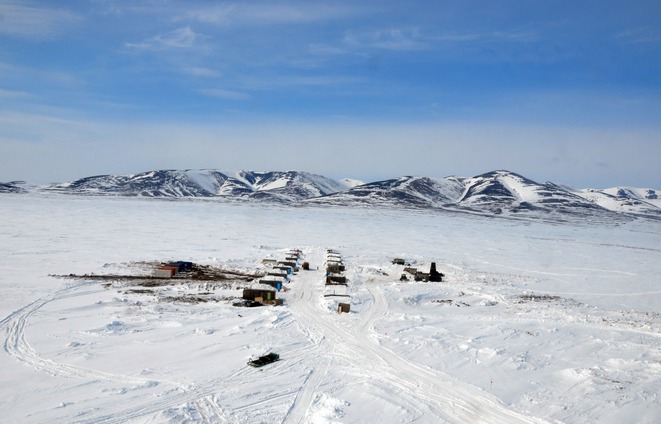
Aerial view of the camp site looking toward the western crater rim.

**Fig. 6:**
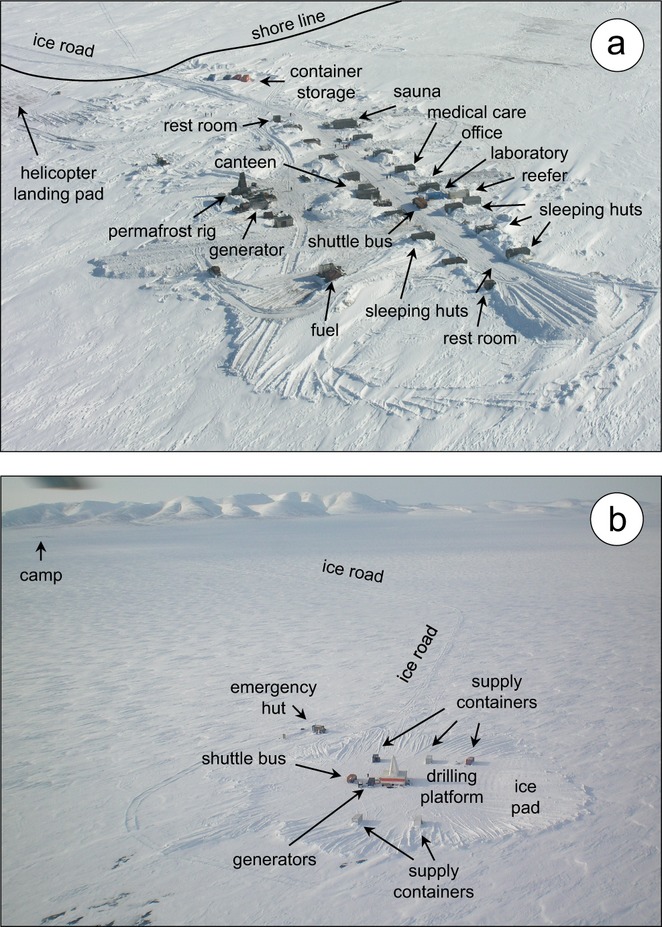
Aerial views of (a) the field camp on the western shore of Lake El'gygytgyn and (b) the drilling platform on the ice pad at ICDP Site 5011-1, from Melles et al. (2011). The camp was designed for up to 36 people with facilities for maintaining two 12 h shifts. The ice pad was first cleared of snow and then artificially flooded with lake water to thicken and strengthen the ice to roughly 2 m. A gas-powered electrical generator fueled all operations. Crew changes along the 7 km ice road to the camp were accomplished by shuttle bus and Russian all-terrain vehicles (“vezdahut”). The ice road was flagged every 25 m for safe travel during whiteouts.

The descriptions of the actual drilling operations follow closely the report by Melles et al. (2011). Because of the remote location of the crater, and the lack of any infrastructure, the project involved a massive logistical undertaking. Figure 4 gives an impression of the routes and distances covered in getting equipment to the crater. During the summer of 2008, most of the technical equipment and field supplies were transported in 15 shipping containers from Salt Lake City, UT, USA, to Pevek, Russia, by ship first to Vladivostok and then on through the Bering Strait to Pevek (Fig. 4a). Two additional containers with equipment were sent from Germany to Vladivostok via the Trans-Siberian Railway. In Pevek, the combined cargo was loaded onto trucks that were then driven with bulldozer assistance across a distance of more than 350 km over winter roads cross country to the El'gygytgyn crater (Figs. 4b and 4c). At the shore of the frozen crater lake, a temporary winter camp was constructed that was designed for up to 36 persons (Fig. 5). The camp consisted of 12 insulated and heated sleeping huts, another hut equipped for medical care, one used as an office, a small canteen, a sauna, and two separate outhouses, built alongside a staging area regularly cleared of heavy snow by snow plows (Fig. 6a). Next to the office hut, a laboratory container was placed that was equipped for whole-core measurements of magnetic susceptibility. In addition, there was a reefer container in which the sediment cores were kept from freezing (as the ambient temperatures could reach −50°C) to prevent destruction of sedimentary structures; no such restrictions applied to the impactite cores. Other camp features included a generator building for electricity supply; storage places for vehicles, fuel, and containers; and a helicopter landing pad.

In total, the project completed one borehole into permafrost deposits in the western lake catchment (ICDP Site 5011-3) and three holes at 170 m water depth in the center of the lake (Site 5011-1). Permafrost drilling at Site 5011-3 was conducted from November 23 until December 12, 2008. Using a mining rig (SIF-650M) that was rented from and operated by a local drilling company (Chaun Mining Corp., Pevek), the crew reached a depth of 141.5 m with a recovery of 91%. After completion of the drilling, the borehole was permanently instrumented with a thermistor chain for future ground temperature monitoring as part of the Global Terrestrial Network for Permafrost (GTN-P) of the International Permafrost Association (IPA), hoping to improve the understanding of future permafrost behavior in the light of contemporary rapid climate change.

**Fig. 7:**
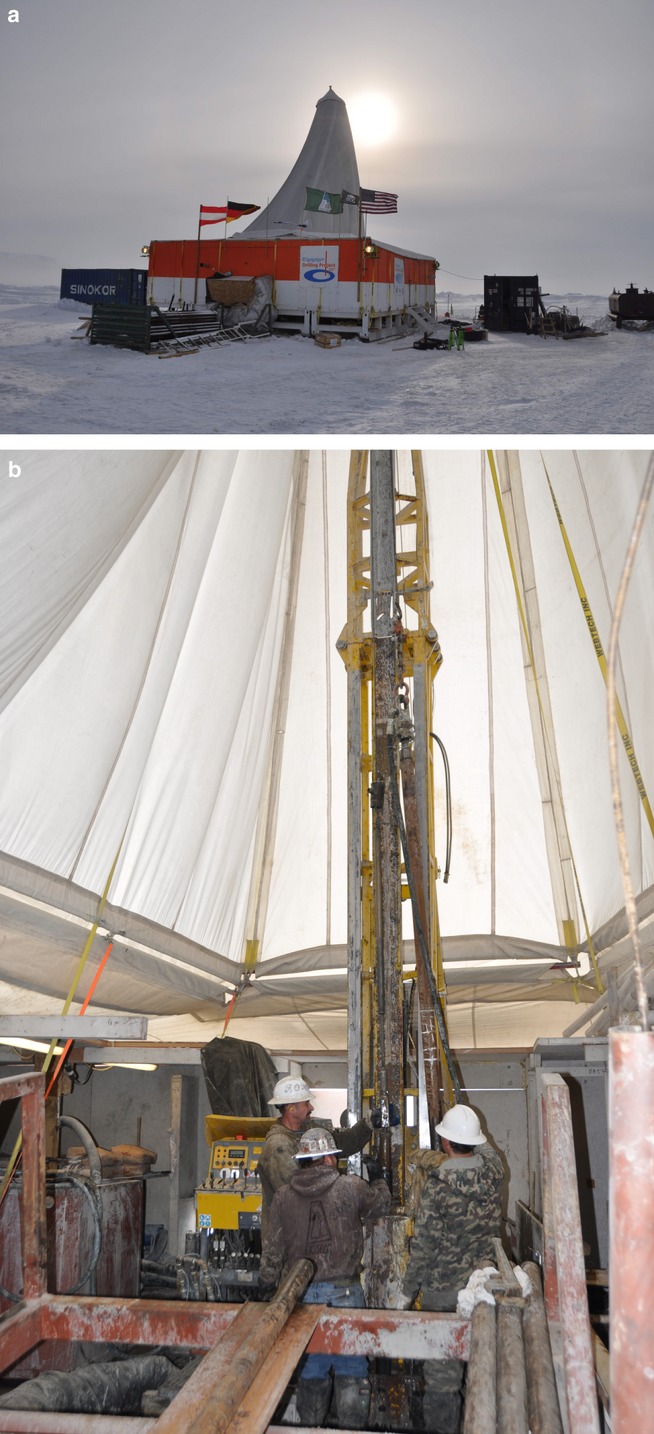
a) The modified GLAD 800 drill rig on a platform contained within a tent to keep the interior above freezing, at ICDP Site 5011-1 at the center of the frozen Lake El'gygytgyn. b) The drill rig in operation within the tent.

In January/February 2009, an ice road between the camp and Site 5011-1 on Lake El'gygytgyn was established based on ice conditions and marked by bamboo poles every 25 m for better orientation during heavy snow storms (Fig. 4c). Subsequently, an ice pad of 100 m diameter at the drill site was artificially thickened to 2.3 m by clearing the snow and pumping lake water onto the ice surface, to allow for lake drilling operations with a 100 ton drilling platform (Fig. 6b). Drilling was undertaken using a lake drilling system similar to the GLAD 800 system that had been employed at Bosumtwi (Koeberl et al. 2007a). The GLAD 800 system used in Russia was developed and adapted for use under extreme cold conditions and was operated by the US consortium DOSECC (Drilling, Observation and Sampling of the Earths Continental Crust). It consists of a modified Christensen CS-14 diamond coring rig positioned on a mobile platform that was weather-protected by insulated walls and a tent on top of the 20 m high derrick (Fig. 7). The system was financed by the major funding agencies of the El'gygytgyn Drilling Project and was permanently imported into Russia, where it remains for further scientific drilling projects.

Drilling at Site 5011-1 was conducted from February 16 until April 26, 2009. The drill plan included the use of casing anchored into the sediment to allow drilling to start at a field depth of 2.9 m below lake bottom (mblb). Holes 1A and 1B had to be abandoned after twist-offs at 147 and 112 mblb, respectively. In Hole 1A, the hydraulic piston corer (HPC) system was used down to 110 mblb, followed by the extended nose corer (EXC) below (details about equipment used are given in Harms et al. 2007). The recovery achieved with these tools was 92%. Similarly, drilling with the HPC down to 100 mblb and with EXC below provided a recovery rate of 98% in Hole 1B. Hole 1C was first drilled by HPC between 42 and 51 mblb, to recover gaps still existing in the core composite from Holes 1A and 1B, and was then continued from 100 mblb. Due to the loss of tools during the twist-offs, further drilling had to be performed with the so-called alien bit corer. The employment of this tool may at least partly explain a much lower recovery of the lake sediments in Hole 1C (recovery rate about 52%), although this could also be due to the higher concentration of gravel and sand in these deeper lake sediments. The recovery increased to almost 100% again at a depth of 265 m, when the tool was changed to a hardrock bit corer (HBC), which has a smaller diameter than the tools employed above. The boundary between lake sediments and impact rocks was encountered at 315 mblb. Further drilling into the impact breccia and brecciated bedrock down to 517 mblb by HBC took place with an average recovery of 76%.

On-site processing of the cores recovered at Site 5011-1 involved magnetic susceptibility measurements with a multisensor core logger (MSCL, Geotek Ltd.) down to a depth of 380 mblb. Initial core descriptions were conducted based on macroscopic and microscopic investigations of the material contained in core catchers and cuttings (lake sediments), and on the cleaned core segments not cored with liners (impact rocks). Additionally, down-hole logging was carried out in the upper 394 m of Hole 1C by the ICDP Operational Support Group (OSG), employing a variety of slimhole wireline logging sondes. Despite disturbance of the electric and magnetic measurements in the upper part of the hole, due to both the presence of metal after the twist-offs at Holes 1A and 1B and some technical problems, these data provide important information on the in situ conditions in the hole (e.g., temperature, natural gamma ray, U, K, and Th contents) and permit depth correction of the individual core segments.

**Fig. 8:**
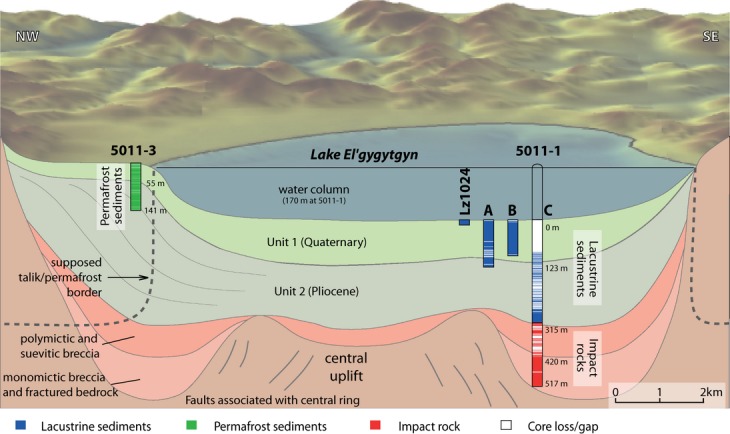
Schematic cross section of the El'gygytgyn basin stratigraphy showing the locations of ICDP Sites 5011-1 and 5011-3 (after Melles et al. 2011). At Site 5011-1, three holes (1A, 1B, and 1C) were drilled to replicate the Quaternary sections. Hole 1C further penetrated the remaining lacustrine sequence and then 200 m into the impact rock sequence. Lz1024 is a 16 m long pilot core taken in 2003 that overlaps between the lake sediment surface and the beginning of the drill cores 1A and 1B at Site 5011-1.

The locations, depth, and schematic lithologies of the drill cores obtained in the drilling project, in comparison with a schematic cross section of the El'gygytgyn crater and lake, are shown in Fig. 8, and a summary of core depths and recovery is given in Table 1.

**Table 1:** Penetration, drilling, and core recovery at ICDP Sites 5011-1 and 5011-3 in the El'gygytgyn crater (all data given in field depth; from Melles et al. 2011)

Site	Hole	Type of material	Penetrated (mblb)	Drilled (m)	Recovered (m)	Recovery (%)
5011-1	1A	Lake sediment	146.6	143.7	132.0	92
	1B	Lake sediment	111.9	108.4	106.6	98
	1C	Total	517.3	431.5	273.8	63
		Lake sediment		225.3	116.1	52
		Impact rocks		207.5	157.4	76
5011-3		Permafrost deposits	141.5	141.5	129.9	91

### Sediment Cores

This brief description follows Melles et al. (2011). Based on the whole-core magnetic susceptibility measurements on the drill cores from ICDP Site 5011-1, the field team was able to confirm that the core composite from Holes 1A to 1C provided nearly complete coverage of the uppermost 150 m of the sediment record in central Lake El'gygytgyn, and that the gap between the top of the drill cores and the sediment surface had been properly recovered by the upper part of a 16 m long sediment core (Lz1024) taken during an earlier site survey in 2003 (cf. Melles et al. 2011). The construction of a final composite core record was completed during core processing and subsampling, which began in September 2009 at the University of Cologne, Germany. The cores were first split lengthwise and both core halves were macroscopically described and documented by high-resolution line scan images (MSCL CIS Logger, Geotek Ltd.). On one core half, color spectra and magnetic susceptibility were measured in 1 mm increments, followed by major and trace element analysis by X-ray fluorescence (XRF) analyses, using an ITRAX Core scanner (Cox Analytical Systems) and X-radiography in steps of 2.0 and 0.2 mm, respectively. Measurements of p-wave velocity and gamma-ray density were then conducted in steps of 2 mm at the Alfred Wegener Institute in Bremerhaven, Germany, before the cores were continuously subsampled after return to Cologne for paleomagnetic and rock magnetic measurements. Subsequently, 2 cm thick slices were continuously sampled from the core composite, excluding deposits from mass movement events, and split into eight aliquots of different sizes for additional biological and geochemical analyses. These aliquots, along with some irregular samples from replicate cores (e.g., for luminescence dating or tephra analyses), were subsequently sent to the sediment science team members responsible for specific studies. In addition, thin sections were prepared from representative sections of the cores to conduct microanalyses of the various lithologies identified during visual core descriptions. After the initial descriptions and sampling procedures have been completed, the remaining, untouched core halves will be shipped to the US National Lacustrine Core Repository (LacCore) at the University of Minnesota, USA, for long-term archiving.

Drilling was very successful because the 315 m-thick lake sediment succession was completely penetrated. The sediments do not seem to include hiatuses due to lake glaciation or desiccation, and their composition reflects the regional climatic and environmental history with great sensitivity. Hence, the record for the first time provides comprehensive and widely time-continuous insights into the evolution of the terrestrial Arctic since Pliocene times. This is particularly true for the lowermost 40 m and uppermost 150 m of the sequence, which were drilled with almost 100% recovery and likely reflect the initial lake stage during the Pliocene and the last approximately 2.9 Ma, respectively. Some first results of the investigations of the sediment cores in terms of paleoclimate studies have been published by Melles et al. (2012) and Brigham-Grette et al. (2013). In particular, the data show that around 3.5 million years ago, immediately after the impact event, summer temperatures at El'gygytgyn were approximately 8 °C warmer than today when pCO_2_ was approximately 400 ppm. Multiproxy evidence suggests extreme warmth and polar amplification during the middle Pliocene, sudden stepped cooling events during the Pliocene-Pleistocene transition, and warmer than present Arctic summers until approximately 2.2 Ma, after the onset of Northern Hemispheric glaciation. The results presented by Brigham-Grette et al. (2013) indicate that Arctic cooling was insufficient to support large-scale ice sheets until the early Pleistocene.

### Permafrost Core

For permafrost research, in November–December 2008 a 142 m-long sediment core was retrieved from the permafrost deposits at ICDP Site 5011-3 in the western lake catchment by the local drilling company Chaun Mine Geological Company (CGE). The core penetrated coarse-grained, ice-rich alluvial sediments with variable contents of fine-grained material. The entire core was completely frozen when recovered. This confirmed modeling results that suggested that the unfrozen talik (a layer of year-round unfrozen ground that occurs in permafrost areas) alongside the lake descends with more or less a vertical boundary until the permafrost base is reached at a depth of a few hundred meters (Fig. 4). The permafrost cores were described and photographically documented after recovery. They were kept frozen in the field and during transport to the ice laboratory (−30 °C) at the Alfred Wegener Institute in Bremerhaven (Germany). There, the cores were cleaned, the documentation was completed, and subsamples were taken from the sediment and ice for ongoing laboratory analyses. Results will be published elsewhere.

### Impactite Core

**Fig. 9:**
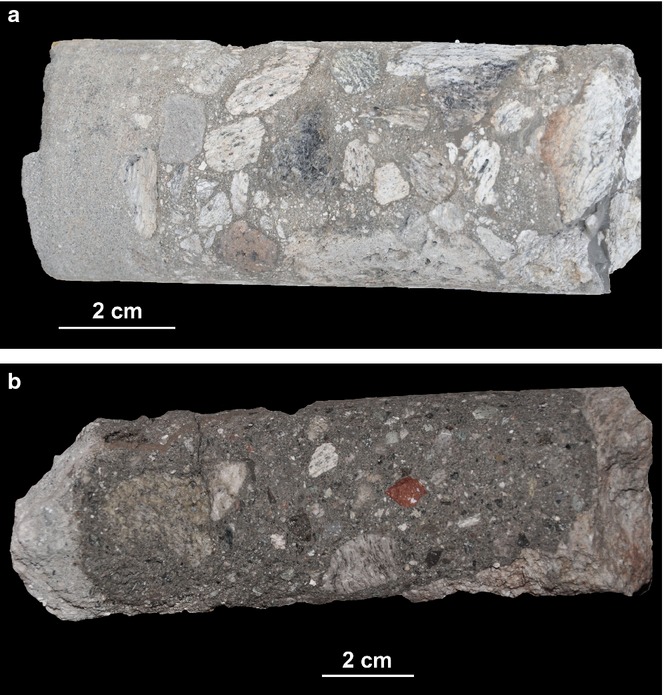
Core segments from the drilling project at the El'gygytgyn impact crater, showing suevitic impact breccia, from (a) about 316 and (b) 319 m below the lake floor, just below the transition from the postimpact lake sediments. The glassy melt rock, which forms during the impact when some of the rock is heated to over 2000 °C, is the dark gray frothy inclusion in the center of the core segment. The cores were photographed by CK in the camp shortly after retrieval.

Core D1c intersected the transition zone between the lacustrine sediments and the main impact breccia sequence at around 315 mblb. The impactite core, described below and the subject of the various papers in this volume, was recovered from 316.75 mblb to a depth of 517.09 mblb. The topmost part of the impactite core segment was recognized even in the field laboratory, immediately after drilling, as a likely suevite (Fig. 9). The core boxes were transported together with the sediment cores from Pevek to St. Petersburg and on to Germany. The impactite core boxes were moved in late 2009 to the Natural History Museum in Berlin, where they were opened, cleaned, photographed, and curated according to ICDP protocol (see Raschke et al. [2013a] for details). The sampling party for the impactite core took place at the Natural History Museum in Berlin on May 15 and 16, 2010. Subsequently, several hundred core samples were prepared and sent to research teams around the world.

#### Impactite Drill Core Stratigraphy

**Fig. 10:**
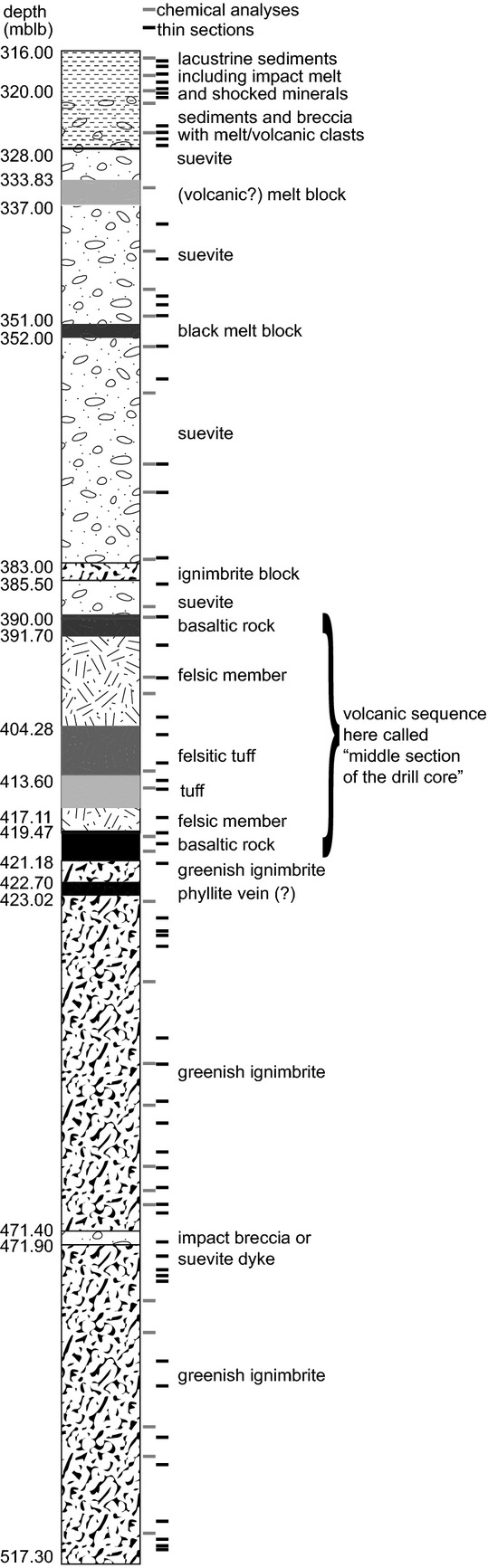
Schematic representation of the simplified drill core litho-stratigraphy (cf. Pittarello et al. 2013), with the samples selected for chemical and petrographic analyses performed at the University of Vienna.

The following description is based on samples studied at the University of Vienna (cf. Pittarello et al. 2013) and differs slightly from complementary efforts by Raschke et al. (2013a) and Wittmann et al. (2013). The studied drill core ranges from 316.80 m to approximately 517 mblb. The whole core can be divided into three main parts: (1) approximately 75 m of polymict lithic breccia/suevite, intercalated with lacustrine sediments in the first 10 m, and containing large melt blocks (up to 40 cm) distributed throughout the profile; (2) approximately 30 m of different volcanic rocks, highly altered, varying from rhyolitic to basaltic lavas, tuffs, and ignimbrites; and (3) approximately 100 m of fractured, welded, rhyo-dacitic ignimbrite, including abundant so-called *fiamme* of pumice, and crosscut by a 50 cm-thick suevite dyke at the depth of 471.40 m. A summary of our lithological classification of the core is shown in Fig. 10.

##### Impact Melt Breccia

This unit can be divided into three subunits: the first two units (from the top) are characterized by the occurrence of lacustrine sediments in the matrix, alternating with impact melt clasts. The overall unit is quite altered, with open fractures, especially at the contact between the impact melt/volcanic blocks and the unconsolidated matrix, where drilling mud penetrated.

**Fig. 11:**
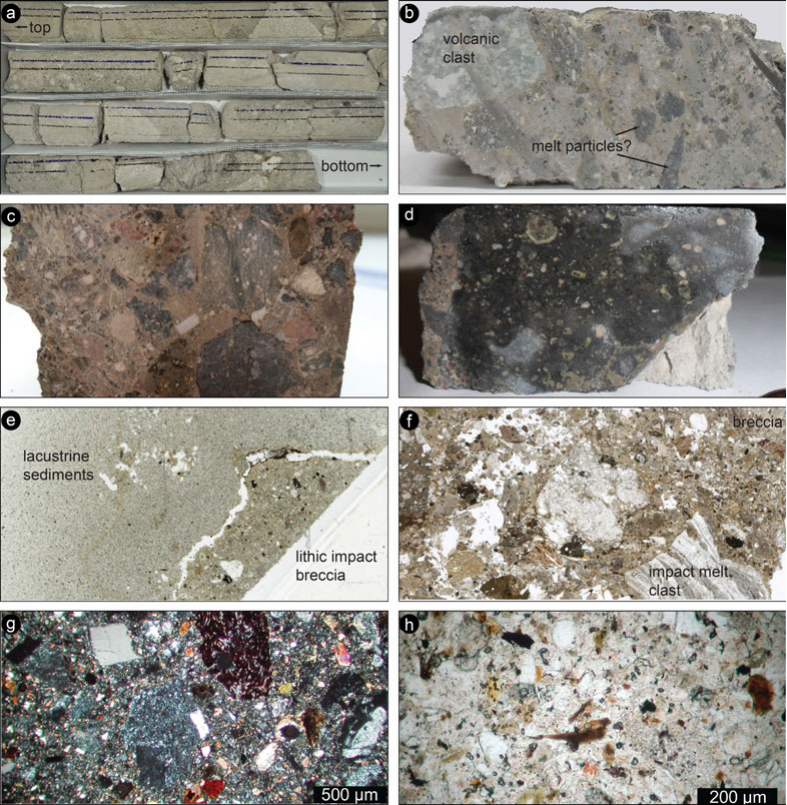
Interval 316.8–320 mblb. a) Box containing the core run 98. The core width is 6 cm. The fine bedding in the lacustrine sediments as well as the impact melt blocks are recognizable (note: the blue and black lines on the core in this and all other core images were applied immediately after core retrieval to indicate the “up” position; with the blue line being on the right when facing up). b) Impact breccia, with possible impact melt (blackish in color) and probably volcanic rock clasts in a grayish matrix, mixed with lacustrine sediments. Sample width 6 cm. Sample 98Q4-W4-8 (317.8 mblb). c) Impact breccia, with poorly sorted clasts of volcanic rocks and impact melt in a reddish matrix. Sample 4 cm wide. Sample 98Q5-W11-15 (318 mblb). d) Impact melt clast, blackish in color and containing small whitish crystals. Sample 4 cm wide. Sample 98Q5-W24-27 (318.4 mblb). Wet surface to enhance the contrast. e) Contact between a fragment of impact breccia and the lacustrine sediments. The contact is open as a result of the sample preparation. Picture 3 cm wide. Thin section scan. Sample 99Q1-W17-19 (319.1 mblb). f) Impact breccia general aspect. Note the extensive porosity (white holes with irregular shape) and the variety of sizes and types of clasts, from impact melt fragments to unshocked volcanic rocks. Image width 3 cm. Thin section scan. Sample 98Q6-W7-11 (318.8 mblb). g) Impact breccia in an enlarged view. Volcanic rock fragments, variously shocked, are recognizable, as well as mineral fragments. Sample 99Q1W17-19 (319.1 mblb). Cross-polarized light microphotograph. h) The matrix of the impact breccia, including angular and rounded mineral fragments and melt particles (dark-brown in color). Sample 99Q1-W17-19 (319.1 mblb). Plane-polarized light microphotograph.

**Fig. 12:**
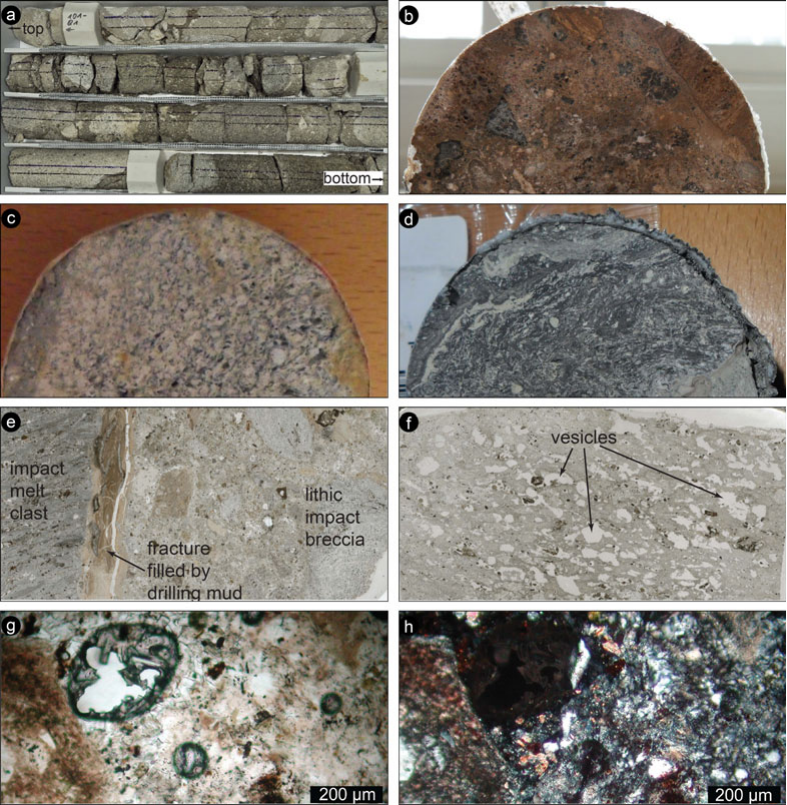
Interval 320–328 mblb. a) Box containing core run 101 (approximately 319–321 mblb). The core width is 6 cm. The lacustrine sediment contribution is reduced in comparison with the core above, but the likely impact melt bodies dominate in this section. Whitish and blackish porous melt boulders, tens of cm long, are visible in the lower rows of the box. b) Sample of impact breccia, with poorly sorted clasts of volcanic rocks and impact melt clasts in a reddish matrix. Sample 6 cm wide. Sample 99Q5-W34-38 (321.3 mblb). c) Sample of likely volcanic rock, grayish in color, showing a layering and few whitish grains. Sample 6 cm wide. Sample 99Q5-W15-17 (321 mblb). d) Sample of impact melt clast, blackish in color and showing a definite internal flow fabric. At the right lower corner of the sample, the contact with the breccia is visible; breccia contains some lacustrine sediments. Sample 6 cm wide. Sample 101Q3-W41-43 (325.8 mblb). e) Contact between a fragment of impact melt (on the left) and the impact breccia (on the right). The contact is marked by a layer of clay, probably from the drilling mud, injected in the open fractures. Picture 3 cm wide. Thin section scan. Sample 101Q6-W11-13 (326.6 mblb). f) Impact melt. Note the extensive vesiculation. The darker portions may represent unmelted material. Picture 3 cm wide. Thin section scan. Sample 101Q8-W41-43 (327.6 mblb). g) The impact breccia matrix. Portion of the impact breccia with a glassy appearance and with rounded vesicles filled by secondary minerals. Sample 99Q3W17-19 (319.1 mblb). Plane-polarized light microphotograph. h) The same area but under cross-polarized light. The glassy matrix is pervasively devitrified. Sample 99Q3-W17-19 (319.1 mblb). Cross-polarized light microphotograph.

**Fig. 13:**
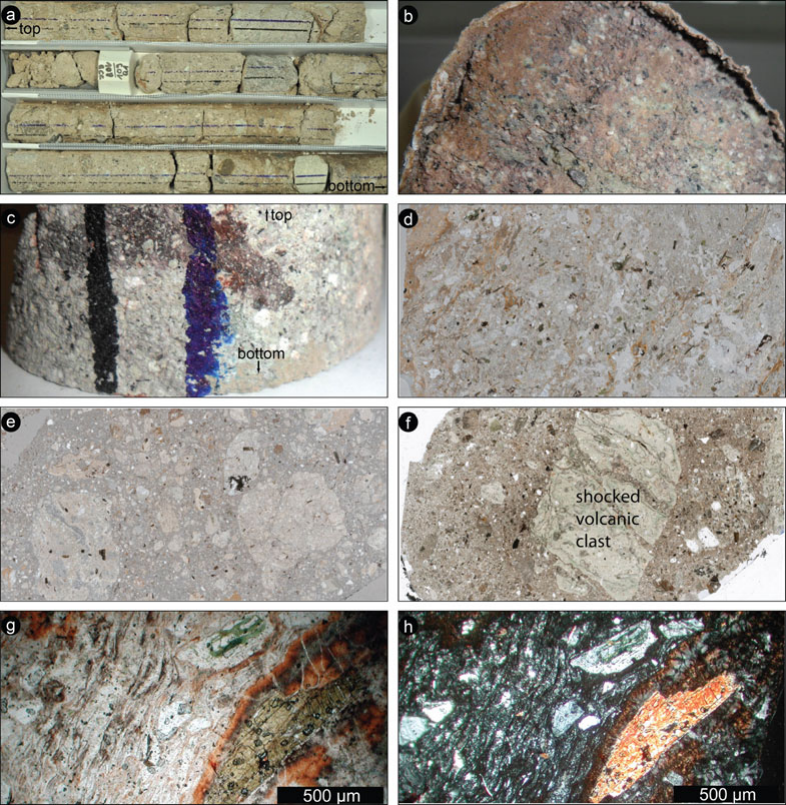
Interval 328–390 mblb. a) Box containing part of the core runs 108 and 109 (approximately 344–350 mblb). The core width is 6 cm. The lacustrine sediment contribution is negligible in this unit, which has a more homogenous appearance. Impact melt bodies are less abundant, whereas in the lower row of the box, a small block of ignimbrite (greenish in color) is visible. b) Sample of impact breccia, with abundant clasts of mm size in a reddish matrix. Sample 6 cm wide. Sample 123Q2-W36-39 (384.4 mblb). c) Sample of ignimbrite (volcanic), with cm-sized pumice fragment. The ignimbrite clearly contains whitish mineral clasts in a grayish matrix. Note the blue and black ink stripes, marking the core orientation (blue on right means “up”). Sample 6 cm wide. Sample 114Q-CC (361.7 mblb). d) Ignimbrite/tuff clast, with strong layering marked by flattened pumice fragments and preferred orientation of the mineral grains. Picture 3 cm wide. Thin section scan. Sample 109Q1-W17-19 (348.6 mblb). e) Impact breccia, with poorly sorted clasts of volcanic rocks in a clastic matrix. Picture 3 cm wide. Thin section scan. Sample 112Q1-W18-20 (355.8 mblb). f) Large rhyolite clast in the impact breccia. Detailed petrographic analysis revealed that the clast is shocked, with plagioclase and quartz phenocrysts containing multiple sets of PDF. Picture 3 cm wide. Thin section scan. Sample 124Q2-W18-20 (387.2 mblb). g) Strong flow fabric in a likely volcanic particle. Sample 109Q1W17-19 (348.6 mblb). Plane-polarized light microphotograph. h) The same area but under cross-polarized light, to note the progress of devitrification in glassy areas and of alteration in phenocrysts. Sample 109Q1-W17-19 (348.6 mblb). Cross-polarized light microphotograph.

1. The interval between 316.8 and 320 mblb (Fig. 11) consists of lacustrine sediments intercalated with impact breccia and impact melt blocks. The lacustrine sediments include fine-grained (sand-size <2 mm) grains, which are equigranular, rounded to subrounded, with many being composed of glass fragments (cf. also Wittmann et al. 2013). In the drill core, lacustrine sediments showing parallel bedding are locally preserved and recognizable. The blocks of impact breccia (suevite, as confirmed by detailed petrographic studies, Pittarello et al. 2013; see also Raschke et al. 2013a) consist of a polymict breccia, with fragments of impact melt, volcanic rocks, and mineral grains in a fine-grained (lower than in the sediments) clastic/glassy matrix. Locally, sediments are mixed in with the matrix. Large impact melt blocks (up to 40 cm) also occur along the drill core. Such impact melt blocks have a variety of colors (from whitish to blackish), but are generally characterized by high porosity (vesiculation), and depending on color, they resemble either volcanic pumice or lava scoria.
2. The interval between 320 and 328 mblb (Fig. 12) is similar to the core section above, but it is marked by an obvious reduction in the lacustrine sediment contribution. The transition is gradual and occurs through a progressive decrease in thickness and abundance of the bedded sediments. A reddish polymict lithic breccia (suevite) progressively becomes the dominant lithology. Such a breccia includes abundant blackish angular melt fragments (up to 2 cm in size), clasts of greenish volcanic rocks, and mineral fragments, suspended in a reddish fine-grained matrix. The core section contains abundant impact melt blocks, similar in size and characteristics to those described in the subunit above, but more frequently observed.
3. The interval between 328 and 390 mblb (Fig. 13) seems more homogenous in terms of lithology. The sediments are totally absent, as well as the impact melt blocks, whereas a reddish breccia dominates. The rock is weakly consolidated and all the samples have to be impregnated with epoxy before proceeding with the thin section preparation. The breccia is a polymict lithic impact breccia, which can locally be classified as suevite, depending on the local occurrence of shocked minerals and impact melt (fact that can be determined only by detailed petrography). The breccia consists of mineral, lithic, and melt fragments in a fine-grained reddish matrix. The melt fragments occur as angular blackish clasts and their sizes (from cm to mm) and abundance seem to decrease progressively through the subunit. Volcanic clasts, a few cm in size, occur in the drill core section.

##### Intermediate Layer—Volcanic Sequence

**Fig. 14:**
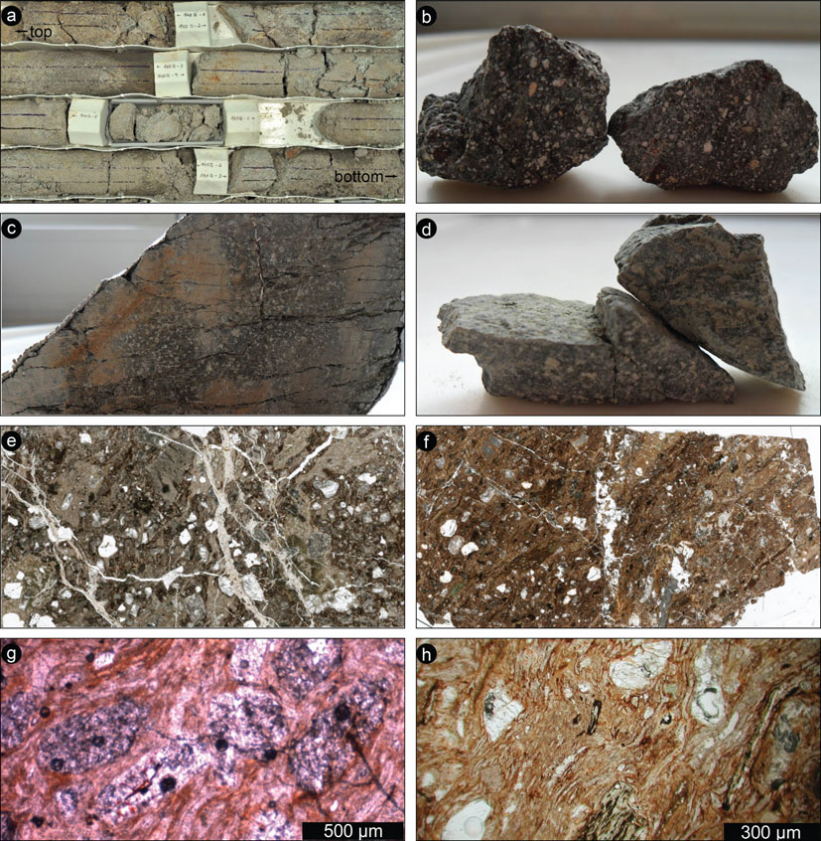
Interval 390–423 mblb: Intermediate layer. a) Box containing part of the core runs 140 and 141 (approximately 416–420 mblb). The core width is 6 cm. The layer includes different lithologies, but the rock is highly altered, making classification difficult. b) Fragments of a layered blackish volcanic rock. Fragments 3 cm wide each. Sample 134Q1-W7-9 (399.6 mblb). c) Sample of a fractured volcanic rock, showing abundant whitish grains in a blackish matrix. Sample 6 cm wide. Sample 142Q2-W1-3 (420.6 mblb). d) Fragments of a greenish volcanic rock, which was classified as basalt by geochemistry. Fragments about 3 cm wide each. Sample 142Q3-W13-15 (420.9 mblb). e) Internal structure of one of the volcanic lithologies in this core section. Subrounded quartz grains are embedded in a brownish matrix, which includes probably pumice lapilli. The sample is crosscut by a network of open fractures. Thin section scan. Sample 137Q1-W5-7 (407.3 mblb). f) Rhyolitic sample with few subrounded quartz phenocrysts in a layered brownish matrix, which shows a strong layering/flow fabric. The sample is crosscut by open fractures, which are discordant with respect to the magmatic foliation. Picture 3 cm wide. Thin section scan. Sample 139Q6-W4-6 (414.8 mblb). g) Strong flow fabric in an andesitic volcanic rock, with abundant altered feldspar grains enveloped by the flowing matrix. Sample 130Q1W15-17 (395.4 mblb). Plane-polarized light microphotograph. h) Felsic volcanic rock with quartz, feldspar, and altered amphibole grains in a glassy welded matrix. Sample 139Q6-W4-6 (414.8 mblb). Plane-polarized light microphotograph.

From 390 to 423 mblb, several volcanic formations follow (Fig. 14). The volcanic sequence is complex and the pervasive alteration makes the classification difficult. Although of similar appearance, the sequence includes subunits with different compositions (from felsic—rhyodacitic, SiO_2_ 70 wt%—to mafic—basalt, SiO_2_ <50 wt%), as revealed by geochemical analysis. The felsic members are generally blackish to reddish in color, with locally recognizable fluidal fabric and porphyritic texture (mm-sized whitish grains). The mafic members are blackish to greenish in color, generally with fluidal fabric, containing abundant whitish grains (phenocrysts). Abundant fractures cut the core section, most of them are open, up to a few mm apart, but a relative displacement between blocks was not observed.

##### Rhyodacitic Ignimbrite

**Fig. 15:**
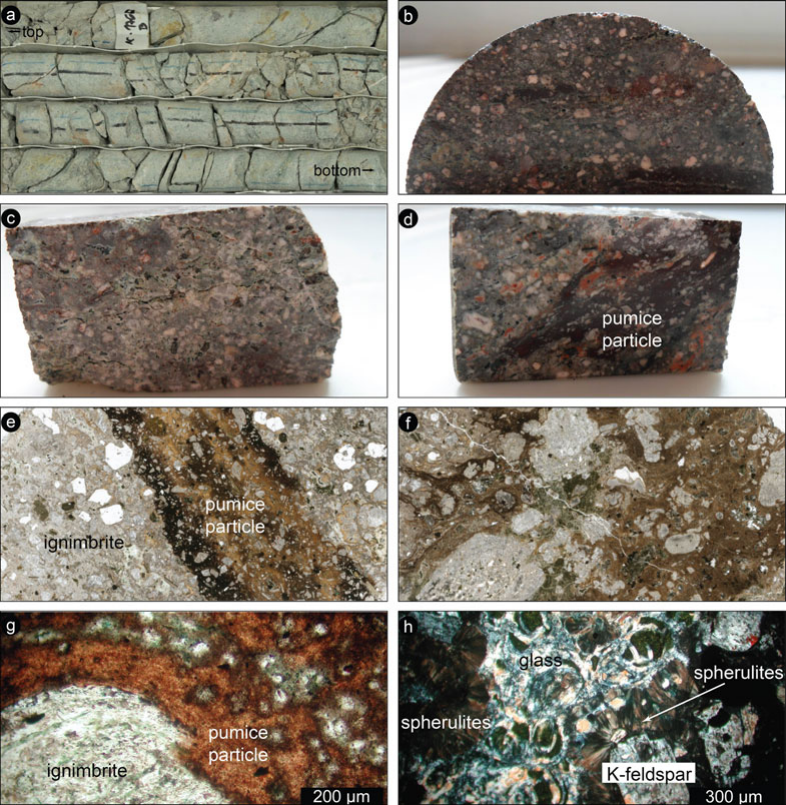
Interval 423–517 mblb: Rhyodacitic ignimbrite. a) Box containing part of the core run 176 (approximately 507–510 mblb). The core width is 6 cm. The core consists of an apparently homogenous greenish ignimbrite, crosscut by fractures and whitish veins filled by both carbonates and zeolites. Fractures and veins are developed with an angle between 15 and 45° with respect to the core axis. b) Cross section of a large pumice clast in the ignimbrite, cut parallel to the flow plane. Note the blackish glassy matrix and the abundant equigranular mineral grains. Sample 6 cm wide. Sample 147Q2-W40-41 (431.8 mblb). c) Pumice-free portion of the ignimbrite. Note the greenish glass preserved in the upper part of the sample. Sample 3 cm wide. Sample 162Q5-W24-26 (470 mblb). d) Ignimbrite containing a large flattened pumice inclusion. Sample is 3 cm wide. Sample 173Q5-W25-27 (501.3 mblb). e) Internal structure of a pumice particle in the ignimbrite. Note the darker color of the matrix and the more abundant feldspar grains at the contact with the host rock, forming the typical “chilled” margins. Thin section scan. Sample 149Q1-W26-28 (435.7 mblb). f) Internal structure of a large pumice particle, with a random distribution of feldspar grains and glass fragments (greenish) in a brownish matrix, characterized by a strong layering. Image width 3 cm. Thin section scan. Sample 164Q3-W35-37 (475.2 mblb). g) Detail of the contact between a pumice particle and host rock matrix. Sample 164Q3-W26-28 (475.1 mblb). Plane-polarized light microphotograph. h) Detail of strongly altered glass (chloritization or devitrification) preserved in the ignimbrite, with the characteristic perlitic fracturing. Note also the extensive development of spherulites at the margins of feldspar grains. Sample 148Q1-W20-30 (433.5 mblb). Cross-polarized light microphotograph.

From 423 to 517 mblb, a single lithology dominates: a rhyodacitic ignimbrite (Fig. 15). This ignimbrite includes abundant welded blackish pumice inclusions (called “*fiamme*” in volcanology, because of their elongated shape). The pumice particles can reach 20 cm in length and 3 cm in thickness. They are aligned, defining an apparent “foliation,” which is determined by the compaction of the pyroclastic deposit. The flattened pumice particles show interfingering contacts with the host and chilled margins, marked by darker intensity of the matrix color and more abundant phenocrysts. The phenocrysts in the pumice particles consist of altered feldspar, whereas quartz is almost absent. The host contains abundant mm-sized whitish grains (quartz and feldspars) in a grayish glassy matrix. Some glass portions are preserved, are generally greenish in color (because of devitrification), and show perlitic fracturing. Locally, a greenish halo of probable glass surrounds the pumice particles. The unit is crosscut by abundant fractures and veins, generally concordant with the magmatic foliation, with a general angle of approximately 45° to the core axis. Locally, conjugate systems of fractures were observed. The veins are generally filled by whitish to reddish or greenish materials, classified as carbonate (likely calcite) or zeolites depending on the reaction to dilute HCl. The overall unit is quite fresh, except for the obvious devitrification of the glassy portions.

The unit is crosscut by an impact breccia dyke between 471.4 and 471.9 mblb. This breccia consists of melt particles and mineral fragments in a glass-bearing clastic, unconsolidated matrix. The contact with the ignimbrite is sharp and no evidence of cataclasis was observed. The breccia was lately better characterized by detailed petrographic studies, revealing the occurrence of shocked minerals (see Pittarello et al. 2013; Raschke et al. 2013a; Wittmann et al. 2013).

## Results of Impactite Studies

Detailed petrographic and geochemical studies of the core samples were performed by three independent groups, in Vienna (Pittarello et al. 2013), Berlin (Raschke et al. 2013b), and Houston/St. Louis (Wittmann et al. 2013). As the three studies involved a different number of samples, and because there is a natural variation in sample characteristics even within a few centimeters of the core, there are differences in the assignment of the exact breccia nomenclature, but the general classification is about the same. In particular, there is still some disagreement regarding the extent to which the uppermost unit is termed a suevite or a reworked suevite.

In a detailed petrographic and geochemical study of the complete drill core, involving over 100 samples for petrography and 35 for geochemistry, Pittarello et al. (2013) found evidence to classify the almost 75 m-thick core section, from about 316 to 390 mblb, beginning with a mixed zone of fallback breccia and lacustrine sediments, as suevite, whereas they assign the remaining part of the core to slightly shocked to unshocked volcanic rocks. These authors noted that the suevite contains abundant melt fragments, as well as shocked minerals. The volcanic rocks that make up polymict and monomict impact breccia comprise a pervasively altered volcanic sequence. Pittarello and co-workers also provide a comparison between the rocks found in the drill core and a representative suite of target rock samples collected at and around the crater. Geochemical studies confirm that the rock types found as parts of the various breccia types are also represented among the target rocks, although the variation in the drill core samples is somewhat limited. As an exception, mafic rocks from the intermediate layer in the drill core cannot be directly correlated with the mafic samples from the target, but Hf-Nd isotopic compositions indicate that the two different types of these rocks represent different stages of the same magmatic evolution.

Raschke et al. (2013a) give an account of the curation and preparation of the impactite cores and discuss the classification of that core according to their observations. These authors concluded that below the zone of reworked impact breccia at the top (316.75–328 mblb), there is a section of what they conservatively refer to as polymict impact breccia (328–390 mblb), followed by two units of variously brecciated volcanic bedrock. The upper bedrock (a unit of various volcanics) and the lower bedrock (rhyodacitic ignimbrite) (391.79–422.71 mblb and 422.71–517.09 mblb). Raschke et al. (2013b) provide detailed petrographic and geochemical observations on their large set of samples that represent the complete impactite core.

Wittmann et al. (2013) performed petrographic and geochemical analyses of a number of drill core samples in comparison with impact melt rocks from the surface and several glass spherules from outside the crater (cf. also Adolph and Deutsch 2009, 2010). Although there are some limited differences between the details of their lithological classifications and those of Pittarello et al. (2013) and Raschke et al. (2013a), due to more limited number of samples and a natural variation in the investigated materials, these researchers still arrive at the same succession of fallback material, suevite, polymict breccia, and monomict breccia as the other authors. Wittmann et al. (2013) quantify the abundance of glassy impact melt shards <1 cm in size in the upper 10 m of suevite to about 1 vol%. Like the other two groups, they also note the finding of glass spherules in the reworked fallout deposit that caps the suevite and is at the transition to lacustrine sedimentation, similar to what was recovered at the top of the Bosumtwi fallback sequence (Koeberl et al. 2007b). Some of the spherules contain Ni-rich spinel and admixtures of an ultramafic component, and this zone also contains a relatively higher abundance of shock metamorphosed lithic clasts. Wittmann et al. (2013) interpret this unit as allochthonous breccia from the vicinity of the central ring uplift of the El'gygytgyn structure.

A main problem in the study of the drill core samples from El'gygytgyn concerns the question how it might be possible to distinguish volcanic melt fragments that are part of the target from those melts and glasses that formed during the impact event. One possibility is the presence of shocked mineral clasts within the glasses, but this opportunity does not always present itself. Recent studies of the cathodoluminescence (CL) properties of volcanic melts and impact melt rocks and glasses from the El'gygytgyn drill core by Pittarello and Koeberl (2013a) indicate that CL parameters might be helpful in distinguishing the two formation processes. Another possibility is the application of quantitative petrography, such as the study of clast size distribution (CSD), as in the study by Pittarello and Koeberl (2013b). Such a technique has been applied to melt rocks in earlier studies, including lunar rocks. These authors show that geometrical characterization provides a reproducible technique for quantitative description of impact lithologies, even though the studied suevite blurs the distinctions due to local variability that averages out on a larger scale. Nevertheless, this method allows the identification of unshocked to slightly shocked volcanic clasts within the suevite.

Pittarello and Koeberl (2013c) studied impact glass samples from the El'gygytgyn structure, to constrain the formation of these glasses and their cooling history. They found that the glasses can be grouped into two types, one that has formed early in the impact process and consists of pure glass (deposited as glass bombs) and a second type that includes composite samples with impact melt breccia lenses embedded in silica glass. These mixed glasses probably resulted from inclusion of unmelted portions into melted portions during ejection and deposition and were probably formed during the crater excavation and modification phase.

Hellevang et al. (Forthcoming) report on laboratory hydrothermal alteration experiments, geochemical modeling, and mineralogical analyses of El'gygytgyn impact melt rock in comparison with two volcanic glass samples (not from the El'gygytgyn region), to better understand the alteration of the El'gygytgyn impact melt and possible relations to the surface of Mars. In their alteration experiments, they found that phases such as cristobalite form; however, as the El'gygytgyn melt rock already contained secondary alteration phases, including zeolites, it was not clear if any additional such phases formed during the experiment.

Goderis et al. (2013) present one of two studies that try to constrain the meteoritic component at El'gygytgyn. In their work, they compare the geochemical composition of impactites from the drill core with that of impact melt rock fragments at the crater surface. They determined siderophile element abundance data and Os isotope ratios and concluded, with the help of mixing calculations taking into account an indigeneous component, that there is evidence for a small (approximately 0.05 wt% carbonaceous chondrite equivalent) meteoritic component at the bottom of a reworked fallout deposit, in a polymict impact breccia, and in some impact melt rock fragments. The exact impactor type could not be derived, but Goderis et al. (2013) suggest, based on siderophile element abundances and ratios of spherule samples that might be part of the uppermost fallback sequence, that an impactor with ordinary chondritic composition is more likely than a primitive achondritic source, even though they do not exclude this possibility completely.

In another study on the meteoritic component within El'gygytgyn impactites, Foriel et al. (2013) note a variation in Cr, Co, and Ni contents in the various breccia and impact glass samples, which do not give a clear signal, but they found that the Cr isotopic composition of an impact glass sample yielded a nonterrestrial ε^54^Cr value of −0.72 ± 0.31 (2 SE). This negative ε^54^Cr differs from values for carbonaceous chondrites (ε^54^Cr of +0.95 to +1.65), but is nearly identical to reported values for ureilites (approximately −0.77), and, within error, similar to values for eucrites (approximately −0.38) and ordinary chondrites (approximately −0.42). Foriel et al. (2013) conclude that the similarity of the El'gygytgyn Cr isotopic data with those of ureilites, and other chemical evidence such as very low Ir contents, suggests that a ureilitic source was involved, or maybe the asteroid could have been an F-type asteroid of mixed composition, similar to the recent Almahata Sitta fall in Sudan.

Finally, an analysis of the physical properties of the drill core from the El'gygytgyn impact structure was performed by Maharaj et al. (2013). These authors studied petrophysical parameters, such as the densities and porosities, and detected structural and textural changes down the drill core, but not changes in lithology. Nevertheless, these parameters can indicate fracturing and brecciation as a result of the impact event, in that they allow the identification of the transition from a consolidated fine-grained matrix structure to a more crystalline structure. These authors suggest that there is a boundary between the differently brecciated rock sections at around 415 mblb. Maharaj et al. (2013) also used paleomagnetic methods to re-orientate the drill core and found that the re-oriented core has natural remanent magnetic components with mainly normal polarity, but also some components with reverse polarity. The magnetic properties suggest that the main magnetic minerals are ferrimagnetic iron-titanium oxides with high titanium contents, as is common for young igneous rocks. These authors note that the variations in magnetic properties are probably caused by differences in the oxidation/reduction state of these ferrimagnetic minerals.

## Conclusions

The El'gygytgyn impact structure, 3.6 Ma old and 18 km in diameter, was excavated in Late Cretaceous siliceous volcanic rocks of the central Chukotka, northeastern Russia. It is the only known terrestrial impact structure formed in siliceous volcanic target and thus enables us to investigate shock metamorphism in such lithologies. The impact structure, filled by a lake 12 km in diameter, was drilled in 2009 during an ICDP drilling project. The drill core penetrated through postimpact sediments, impactites, and the fractured igneous basement. The impactite portion of the core was recovered from 316.08 to 517.30 m in depth below the lake bottom.

The main rock types of the crater basement are ignimbrite, tuff, and lava of rhyolitic to dacitic composition; rarely basaltic and andesitic compositions were analyzed. The simplified stratigraphy of the core is: (a) 316–390 m—impact breccia including volcanic and impact melt clasts that locally contain shocked minerals, in a fine-grained clastic matrix; (b) 385–423 m—a volcanic sequence including both felsic (likely felsic tuffs) and mafic (basalt) members; (c) 423–517 m greenish rhyo-dacitic ignimbrite, with abundant (volcanic) melt particles, quartz-free and elongated parallel to flattening direction. This latter formation is crosscut by abundant fractures locally filled by carbonate, silicate, and clay veins. Over the whole length of the impactite core, the abundance of shock features decreases rapidly from the top to the bottom of the studied core section, being almost absent in the lower brecciated volcanics.

A comparison between the similar sized Bosumtwi and El'gygytgyn impact craters is quite interesting, despite the difference in target rocks. Initial expectations of large amounts of impact melt within either of those craters were not confirmed. A large variety of stratigraphic, petrographic, geochemical, isotopic, and petrophysical analyses were made on the impactite core segment by several research teams and are reported in a series of companion papers to this introduction and overview.
